# Expression of adiponectin in the subchondral bone of lumbar facet joints with different degrees of degeneration

**DOI:** 10.1186/s12891-017-1786-8

**Published:** 2017-11-03

**Authors:** Qi Lai, Yuan Liu, Leitao Huang, Xuqiang Liu, Xionglong Yu, Qiang Wang, Runsheng Guo, Jianghao Zhu, Hanxiong Cheng, Min Dai, Bin Zhang

**Affiliations:** 1 Department of Orthopedics, The First Affiliated Hospital of Nanchang University, 17 Yongwai Street, Nangchang, Jiangxi 330006 China; 2 Artificial Joints Engineering and Technology Research Center of Jiangxi Province, Nanchang, Jiangxi 330006 China

**Keywords:** Adiponectin, Facet joint, Osteoarthritis, Subchondral bone, Cartilage, Bone degeneration

## Abstract

**Background:**

Osteoarthritis research has been most commonly performed in the setting of the articular cartilage of the knee. To the best of our knowledge, no studies have evaluated the role of adiponectin in osteoarthritis of the lumbar facet joint (FJOA). Therefore, in this study, we explored whether adiponectin was expressed in the lumbar facet joints and evaluated the role of adiponectin in FJOA.

**Methods:**

We enrolled patients who underwent lumbar computed tomography (CT) and magnetic resonance imaging (MRI) at the Orthopedic Department of the First Affiliated Hospital of Nanchang from May 2015 to June 2016. Lumbar facet joints were obtained from 135 patients at the time of lumbar fusion surgery and divided into three groups according to the Weishaupt grade. Cytokine levels in the subchondral bones were evaluated by enzyme-linked immunosorbent assays (ELISAs), and adiponectin levels were determined by immunohistochemistry, western blotting, and quantitative polymerase chain reaction (qPCR).

**Results:**

By ELISA, adiponectin levels were examined in the subchondral bone for lumbar facet joint, and adiponectin was found to be negatively correlated with BMI in 52 patients (*p* < 0.001, *r* = −0.861). By immunohistochemistry analysis, adiponectin was found to be expressed in the subchondral bone of the lumbar facet, whereas the cartilage area was negative for adiponectin expression. Immunostaining intensity and area was related to the degeneration of the lumbar facet joint, and, in our research, considerably decreased staining intensity and area were observed in more severely degenerated lumbar facet joints. Furthermore, the expression of adiponectin was also reduced in degenerated lumbar facet joints, and the level of decline corresponded to degeneration detected by western blotting and qPCR analysis (*n* = 27, *p* < 0.0001).

**Conclusions:**

Adiponectin expression was observed in the subchondral bone of the lumbar facet joint and decreased as the degree of degeneration increased. Thus, the results of this study provide new insights into the relationship between adiponectin and osteoarthritis.

**Electronic supplementary material:**

The online version of this article (10.1186/s12891-017-1786-8) contains supplementary material, which is available to authorized users.

## Background

Lumbar facet joint osteoarthritis (FJOA) is present in approximately 40% of patients with low back pain [1–3]. Therefore, in addition to studies on disc degeneration, studies of FJOA are essential for the prevention of low back pain. The lumbar facet joint is a synovial joint composed of cartilage, synovium, and an articular capsule, and its characteristics are similar to those of other synovial joints, such as the knee [4–6]. However, most studies of osteoarthritis (OA) have focused on the knee joint [7–10]. Some studies have shown that knee OA may be a disease of the entire joint, including articular cartilage, subchondral bone, meniscus, ligament, and neuromuscular groups [11, 12]. Moreover, the subchondral bone plays a major role in knee joint degeneration. However, few studies have evaluated lumbar facet OA or the subchondral bone by basic research, with scholars instead focusing more on clinical research areas [13, 14].

In 2015, we reported a predictive experiment involving cytokine screening in five cases of lumbar facet joint specimens by RayBio® Human Inflammation Antibody Array G3. The screening results showed that interleukin (IL)-1β, TNF α and β, leptin, adiponectin, Chemokine (CCL)-11, CCL-24, colony stimulating factor (CSF)-2, CSF-3, intercellular adhesion molecule (ICAM-1), interferon (IFNg), IL-1–16, monocyte chemotactic protein (MCP), macrophage inflammatory protein (MIP), and others were detected in five cases of lumbar facet joint specimens. The authors discovered that adiponectin showed a strong positive reaction and high expression. Adiponectin is a cytokine secreted by adipose tissue [15, 16] and is abundantly expressed in the circulation in three different molecular forms: trimer, hexamer, and high-molecular-weight (HMW) species [17]. Early studies have suggested that adiponectin plays a key role in the control of energy homeostasis because its plasma levels are inversely correlated with body mass index (BMI), intra-abdominal fat, and indices of insulin resistance [18, 19]. Therefore, adiponectin may be inversely correlated with OA because BMI is a risk factor of OA. In addition, Berner et al. [20] discovered that adiponectin and its receptors are expressed in bone-forming cells in mice and that adiponectin may affect bone metabolism. Moreover, Berner et al. [20] suggest that fatty acids have a regulatory effect on adiponectin mRNA expression, and fatty acids provide increased energy supply to cells and enhance adiponectin expression in osteoblasts. Coincidentally, Francin and Presle et al. [21] reported that the elevated level of adiponectin found in chondrocytes from patients with knee OA might contribute to matrix remodelling during OA; notably, the regulation of bone metabolism by adipokines is largely unknown, and the observed expression and secretion of adiponectin by bone-forming cells serves to add more complexity, as well as redundancy, to this intriguing issue. Therefore, the specific role of adiponectin in FJOA is still unclear and controversial.

Accordingly, in this study, we aimed to evaluate whether adiponectin was expressed in the subchondral bone of lumbar facet joints and to explore the role of adiponectin in FJOA.

## Methods

### Clinical samples and data

**Fig. 1:**
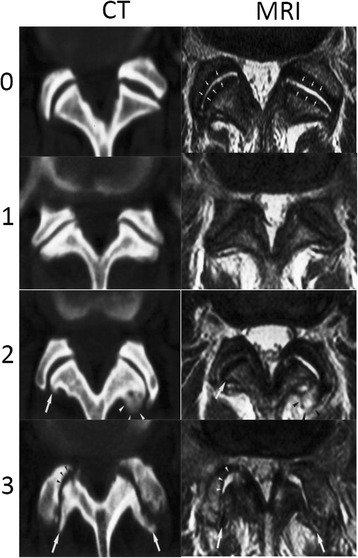
The Weishaupt grade of CT and MRI images of the lumbar facet joint: Grade 0, normal facet joint space (2–4 mm width); Grade 1, narrowing of the facet joint space (<2 mm) and/or small osteophytes, and/or mild hypertrophy of the articular process; Grade 2, narrowing of the facet joint space and/or moderate osteophytes, and/or moderate hypertrophy of the articular process, and/or mild subarticular bone erosions; and Grade 3, narrowing of the facet joint space and/or large osteophytes, and/or severe hypertrophy of the articular process, and/or severe subarticular bone erosions, and/or subchondral cysts

Subchondral bone was obtained from the lumbar facet joints of 135 patients (median age: 51.38 years; range: 16–77 years) at the time of lumbar fusion surgery. Additionally, BMI data of 60 of the patients (BMI: 19.4–33.8 kg/m^2^; mean 23.85 kg/m^2^),which were part of the 135 patients, were collected to examine the release of adiponectin from the subchondral bone of the lumbar facet joint in relation to BMI. Thirty-two normal facet joint specimens were obtained from L3-L5 vertebral fracture decompression or fusion surgeries, and 103 degenerative samples were obtained from L3-S1 single segment lumbar disc herniation fusion surgeries (60 for ELISAs, 48 for immunohistochemical analysis, and 27 for qPCR and western blotting). Samples were stored at −80 °C after surgery until use. All samples were obtained from the Orthopedic Department of the First Affiliated Hospital of Nanchang University from May 2015 to June 2016. All lumbar facet joints were grouped according to the Weishaupt [22] grade, as determined by computed tomography (CT) and magnetic resonance imaging (MRI), as follows: 0, normal; 1, slight degeneration; 2, moderate degeneration; 3, severe degeneration (Fig 1). Lumbar facet joints were divided into three groups (normal group [NG]: grade 0; degeneration group [DG]: grades 1 or 2; severe degeneration group [SDG]: grade 3) according to computed tomography (CT) and magnetic resonance imaging (MRI) results based on Weishaupt grading because of subjective interference in grades 1 and 2 [23].

Inclusion criteria were as follows: L3-L5 vertebral burst fracture or L3-S1 lumbar disc and facet joint degeneration; no history of spinal surgery; underwent lumbar CT and MRI examination. The exclusion criteria were as follows: lumbar spondylolisthesis, scoliosis, lumbar spine infection, spinal tumors and other spine-related diseases; diabetes, hypertension, and other relevant medical history; history of smoking or alcoholism; psychological disorders, mental disorders, or drug use and other history. The study protocol was approved by the Institutional Review Board of the First Affiliated Hospital of Nanchang University, and written informed consent was obtained from all study participants.

### Screening of chemical factors by ELISA

Lumbar facet joint samples were obtained from 60 patients (NG, *n* = 11; DG, *n* = 34; SDG, *n* = 15; BMI: 19.4–33.8 kg/m^2^; mean 23.85 kg/m^2^). After washing the lumbar facet joint in sterile phosphate buffered saline (PBS), full-depth standardized subchondral bones (120 mg for each specimen) were collected using a scalpel. The subchondral bone (30 mg) was ground with liquid nitrogen into a powder, which was mixed with 1000 μL tissue lysis solution. Then, tissue debris was removed by centrifugation at 12,000×*g* at 4 °C for 15 min. In addition, 800 μL supernatant samples were obtained. ELISA kits (RayBiotech, Inc.) were used to quantify IL-1β, TNF-α, leptin, and adiponectin in supernatants after centrifugation. Briefly, eight steps were performed according to the instructions of ELISA kits. Finally, the absorbance of the sample was measured at 450 nm. The mean absorbance for each set of duplicate standards, controls, and samples was calculated, and the average zero standard optical density was subtracted. The standard curve was plotted on log-log graph paper, with the standard concentration on the x-axis and the absorbance on the y-axis.

### Assessment of adiponectin expression by immunohistochemistry

The lumbar facet joint samples were obtained from 48 patients (NG, *n* = 12, median age: 45.56 years; DG, *n* = 12, median age: 47.33 years; SDG, *n* = 24, median age: 58.75 years) were fixed in 4% buffered paraformaldehyde for 48 h and decalcified with buffered ethylenediaminetetraacetic acid (EDTA, 20%; pH 7.4); the buffer was replaced every 3 days until the pin could be easily pierced. After dehydration and embedding in paraffin, sections were cut to a thickness of 4 μm. Samples were heated for 90 min, deparaffinized with dimethylbenzene, and dehydrated in a graded ethanol series (85%, 90%, and 100%). The sections were then subjected to antigen retrieval using microwave heating in citrate buffer (pH 6.0) for 12 min, and endogenous peroxidases were blocked with 3% H_2_O_2_ for 8 min. Serial sections from each case were stained with hematoxylin, hydrochloric acid alcohol, and carbonic acid aluminium and then washed three times with PBS. The sections were incubated with anti-adiponectin antibodies (1:400) or PBS alone as a control at 4 °C for 12 h, followed by washing and incubation with biotinylated secondary antibodies at 37 °C for 30 min. The immunoreaction was finally visualized with diaminobenzidine (DAB) and counterstained with hematoxylin. Human adipocyte tissue was used as a positive control. Specimens were evaluated under a light microscope by an expert pathologist and scored based on a semiquantitative approach of the percentage of positive subchondral bone (0–100%) and the staining intensity (0, negative; 1, weak; 2, moderate; 3, strong) in each subchondral bone sample.

### Assessment of adiponectin expression by western blotting

Lumbar facet joint samples were collected from 27 patients (NG, *n* = 9, median age: 44.44 years; DG, *n* = 9, median age: 45.33 years; severe SDG, *n* = 9, median age: 57.22 years). Protease inhibitor (10 μL) and 990 μL RIPA buffer were added to the 50 mg samples, and the samples were then ground on ice and mixed on a rotator for 30 min at 4 °C. Tissue debris was removed by centrifugation at 12,000×*g* at 4 °C for 15 min, and protein concentrations were measured using a Bio-Rad Protein Assay kit (Bio-Rad, Hercules, CA, USA). Protein samples (20 μg) were subjected to sodium dodecyl sulfate polyacrylamide gel electrophoresis and electrophoretically transferred to polyvinylidene difluoride membranes. The membranes were sequentially blotted with the primary antibody (anti-adiponectin 19F1; ab22554; Abcam, Cambridge, UK) and secondary antibody and developed using enhanced chemiluminescence.

### Assessment of adiponectin expression by qPCR

Subchondral bone (50 mg) was ground with liquid nitrogen into a powder, and total RNA was extracted using TRIzol reagent (Transgen Biotechnology Co., Beijing, China), per the manufacturer’s instructions. Total RNA (1 μg) was employed to prepare cDNA via reverse transcription using a PrimeScript RT Reagent kit with gDNA Eraser (Perfect Real Time; DRR047A; TakaRa, Shiga, Japan). cDNA samples (2.8 μL per 20 μL reaction) were analysed for genes of interest and reference genes (h-actin and adiponectin). qPCR was performed using SYBR Premix Ex TaqTM II (Tli RNaseH Plus; DRR820A; TakaRa) with an ABI StepOnePlus system (Applied Biosystems, Inc., Foster City, CA, USA). The cycling profile was as follows: denaturation at 94 °C for 30 s; 40 cycles of annealing at 60 °C for 15 s, primer extension at 72 °C for 60 s, and denaturation at 95 °C for 30 s; and a final extension for 2 min. The comparative RQ value method was used to determine fold changes in expression using β-actin as a control. The following primers were used: H-actin-285, F5′-AGCGAGCATCCCCCAAAGTT-3′ and R5′-GGGCACGAAGGCTCATCATT-3′; ADIPOQ (214 bp), F5′-CATGCCCATTCGCTTTACCA-3′ and R5′-GGAGGCCTGGTCCACATTAT-3′. The primer sequence was selected for high match similarity to *Homo sapiens* adiponectin gene through NCBI primer blast.

### Statistical analysis

Statistical analysis was conducted with GraphPad Prism 5.0 software (San Diego, California, USA). To study the release of adiponectin from the subchondral bone of the lumbar facet joint in relation to the BMI, Pearson correlation analysis was performed on adiponectin content data determined by ELISA and the BMI measurements of patients. Additionally, data analysis of the immunohistochemistry, western blotting, and qPCR experiments was performed using one-way ANOVA with post hoc examination of significant main effects using the Dunnett method. Data are presented as the mean ± SD, unless stated otherwise. A *p* value less than 0.05 was considered significant for differences and correlations.

## Results

### Expression of IL-1β, TNF-α, leptin, and adiponectin in lumbar facet joints

**Fig. 2:**
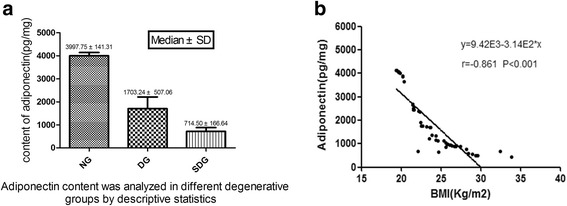
**a** The mean value of the adiponectin concentration was 3997.75 ± 141.31 pg/mg in the normal group, 1703.24 ± 507.06 pg/mg in the degenerative group, and 714.50 ± 166.64 pg/mg in the severe degenerative group by descriptive statistics. **b** Adiponectin was negatively correlated with the BMI of 52 patients (8 cases having extreme values were removed), *p* < 0.001 and *r* = − 0.861

First, we evaluated the expression of several cytokines in lumbar facet joint specimens using ELISA. Since IL-1β, TNF-α, and leptin were detected in only a few specimens, they were not included in the statistical analysis. For the adiponectin content data, extreme values (8 case of specimens) were removed and were analysed via descriptive statistics. The result showed that adiponectin was detected in all facet joint specimens, and adiponectin content was different in the different degeneration groups (Fig. 2a). In addition, adiponectin was negatively correlated with BMI in 52 patients (correlation coefficient: *p* < 0.001, *r* = −0.861; Fig. 2b).

### Qualitative analysis of adiponectin by immunohistochemistry

**Fig. 3:**
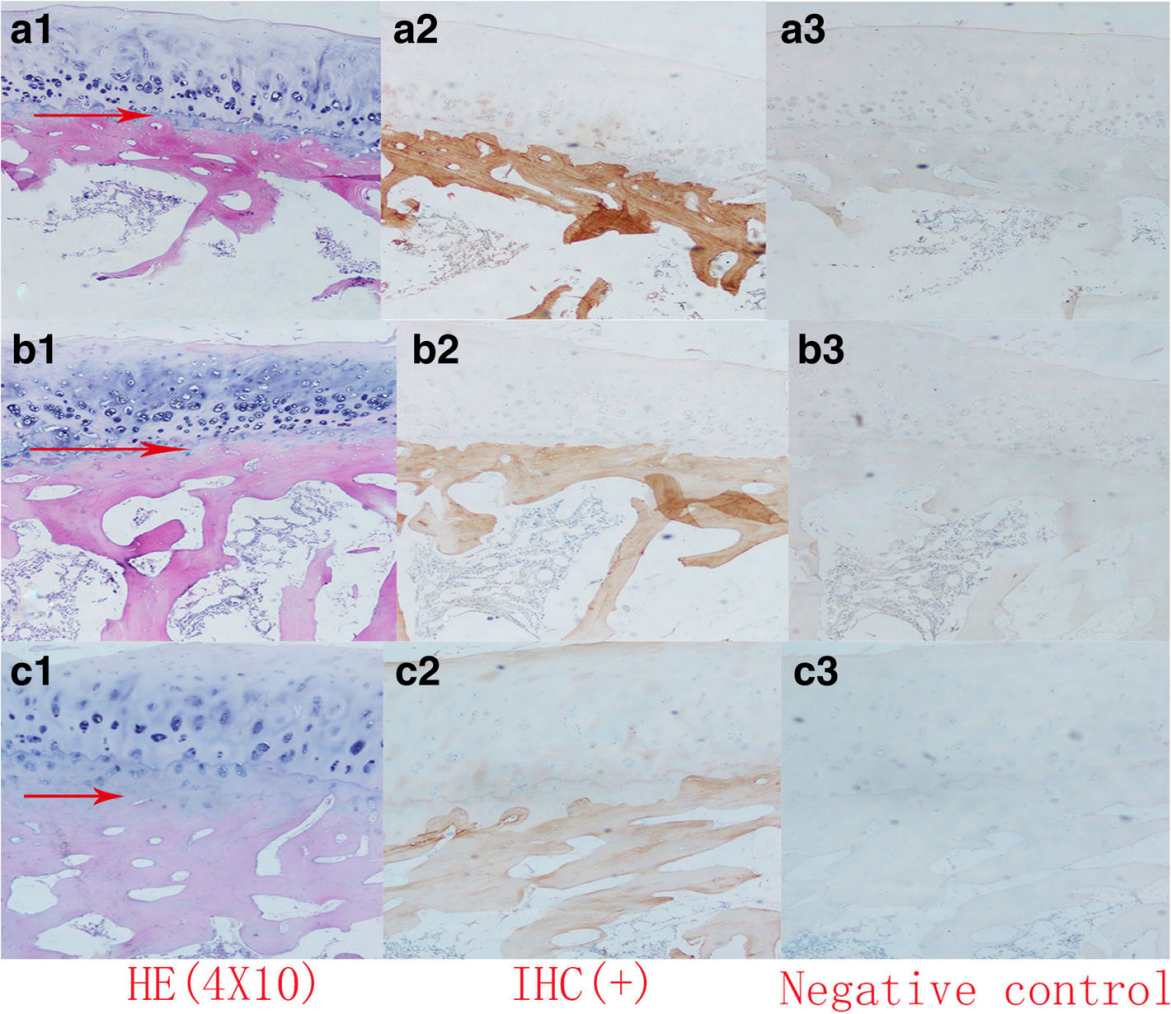
**a1** Hematoxylin-eosin(HE) staining of the lumbar facet joint in the normal group showed that that the cartilage and subchondral bone boundary were clear and the chondrocyte arrangement rules (a: NG, normal group). **a2** Immunohistochemistry of the lumbar facet joint in the normal group showed strong positive staining in the subchondral bone area, whereas the cartilage area showed negative staining. **a3** PBS, instead of a primary antibody, was used as a negative control in the immunohistochemical analysis. **b1** HE staining of the lumbar facet joint in the degeneration group showed that the cartilage and the subchondral bone area had blurred boundaries and irregular chondrocyte arrangement. (b: DG, degenerative group). **b2** Immunohistochemistry of the lumbar facet joint in the degeneration group showed that the subchondral bone area stained positive, and the cartilage area stained negative. **b3** PBS, instead of a primary antibody, was used as a negative control in the immunohistochemistry. **c1** HE staining of the lumbar facet joint in the severe degeneration group showed that chondrocytes extended through the cartilage line and that chondrocytes were reduced in number and disordered (c: SDG, severe degeneration group). **c2** Immunohistochemistry of the lumbar facet joint in the severe degeneration group showed that the subchondral bone area staining was weakly positive, whereas the staining of the cartilage area was negative. **c3** PBS, instead of a primary antibody, was used as a negative control in the immunohistochemical analysis

**Fig. 4:**
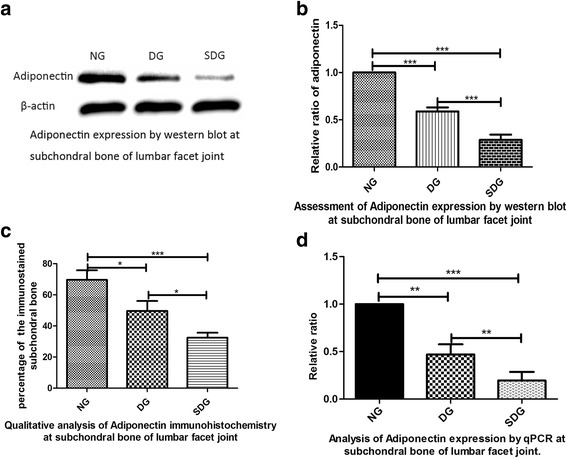
**a** The expression of adiponectin in the subchondral bone area of the lumbar facet joints by western blotting. The results showed that adiponectin levels were significantly reduced in correlation with the degree of lumbar facet joint degeneration. Representative results from three experiments are shown. (NG: normal group; DG: degenerative group; SDG: severe degenerative group). **b** The grey value of the protein band was analysed by one-way ANOVA. The results showed significant differences in the three group. (NG: normal group, *n* = 9; DG: degenerative group, *n* = 9; SDG: severe degenerative group, *p* < 0.0001). **c** Determination of the immunohistochemical staining percentages was performed in the three groups by one-way ANOVA. The results showed that *p* < 0.0001 in NG compared to SDG and *p* < 0.05 in NG compared to DG and DG to SDG. **d** Adiponectin levels were significantly reduced in correlation with the degree of FJOA by qPCR. Determination of RQ values was performed by one-way ANOVA. The results showed that *p* < 0.0001 in the NG group compared to the SDG group, whereas *p* < 0.001 in NG compared to DG and DG to SDG. Additionally, * represents *p* < 0.05, ** represents *p* < 0.001, and *** represents *p* < 0.0001

Next, we examined whether adiponectin was expressed in the subchondral bone of the lumbar facet joint by immunohistochemistry. We evaluated adiponectin expression in 48 specimens (NG, 12; DG, 12; SDG, 24) by immunohistochemical staining. The results showed that adiponectin was expressed in the subchondral bone but not in the cartilage (Fig 3). Compared to the percentage of immunohistochemical staining in the normal group, significant differences (*p* < 0.0001) were identified in the severe degeneration group by one-way ANOVA (Fig. 4c). Compared to the percentage of immunohistochemical staining in the normal group, a statistically significant difference (*p* < 0.05) was observed in the degeneration group by one-way ANOVA (Fig. 4c). In addition, compared to the percentage of immunohistochemical staining in the degeneration group, a significant difference (*p* < 0.05) was also found in the severe degeneration group by one-way ANOVA (Fig. 4c).

### Quantitative analysis of adiponectin expression by western blotting and qPCR in the subchondral bone of the lumbar facet joint

Finally, in order to test our hypothesis that adiponectin plays an important role in FJOA, we further evaluated adiponectin expression by western blotting and qPCR. Western blot analysis demonstrated that adiponectin expression was significantly higher in the normal group than in the degenerative and severe degeneration groups by one-way ANOVA (*p* < 0.0001; Fig. 4a and b). In addition, adiponectin mRNA levels were significantly lower in the degenerative and severe degeneration groups as compared with those in the normal group when normalized to the expression of β-actin by one-way ANOVA (*p* < 0.0001; Fig. 4d).

## Discussion

As a part of the three-column structure of vertebrae, facet joints play a key role in maintaining the stability of spinal motion, particularly in the lumbar area [24]. Therefore, most doctors are concerned with the biomechanical and mechanical factors affecting lumbar facet joints. However, the pathogenesis of FJOA has not been fully elucidated. Accordingly, in this study, we evaluated the expression of adiponectin in lumbar facet joints. Our results showed that adiponectin expression was significantly downregulated with increasing degeneration of the lumbar facet joint.

Adiponectin is an adipocyte factor specifically secreted from fat cells. Adiponectin is composed of 244 amino acids; the N-terminus contains one secretory signal sequence, and the C-terminus contains a spherical egg white function threshold. Previous studies have shown that adiponectin is involved in glucose homeostasis, insulin sensitivity, and vascular inflammatory diseases [25, 26]. Adiponectin in human peripheral blood circulation has three different types of polymer, including low- molecular-weight trimers (LMW), a middle-molecular-weight hexamers (MMW), and high numbers of monomers that compose high-molecular-weight multimers (HMW). Yamauchi et al. [27] reported that the affinity of adiponectin varies between different subtypes and receptors and that the adiponectin monomer, AdipoR1, and AdipoR2 have higher affinity, while MMW and HMW are mainly associated with T-cadherin. Therefore, the biological effects of the different types of adiponectin polymers may vary considerably. In research on metabolic and cardiovascular disease, HMW adiponectin was found to induce proinflammatory cytokine production [28], whereas LMW adiponectin was shown to inhibit the release of inflammatory factors [29]. Additionally, in studies focusing on arthritis, the adiponectin monomer and OA were negatively correlated, and HMW adiponectin was not associated with the degree of OA [30]. Furthermore, Kang et al. [31] showed that adiponectin is a potential catabolic mediator of OA in vitro. Therefore, the relationship between adiponectin and OA is of considerable interest.

In this study, the results showed that adiponectin expression was significantly higher than IL-1β, TNF-α, and leptin expression in the lumbar facet joints by ELISA and that adiponectin was negatively correlated with BMI. These findings support a model in which adiponectin is negatively correlated with FJOA. Furthermore, western blotting and qPCR confirmed the down regulation of adiponectin in degenerative joints compared with that in normal joints. Therefore, our findings suggest that degeneration of the lumbar facet joint may be significantly associated with adiponectin expression. However, the specific mechanism is not clear. Berner et al. [20] found that adiponectin and its receptors are expressed in bone-forming cells of the juvenile mouse mandible and that adiponectin promotes the metabolism of osteoblasts in bone. Chen et al. [32] provided evidence for the protective role of adiponectin in knee OA. The study found that adiponectin can activate p38 mitogen-activated protein kinase (p38MAPK) pathway, involved in the pathogenesis of OA [33, 34]. However, few studies have evaluated the subchondral bone, which can play an important role in FJOA. The subchondral bone of the lumbar facet is composed of the cortical plate, trabecular bone, bone trabecula, and vascular lacuna. Small veins, small arteries, and sinusoidal ducts enter the subchondral bone area and then into the cartilage and cartilage radiation layer through the cortical plate, providing nutrients to the deeper cartilage [35, 36]. Orth and Cucchiarini [37] demonstrated that the subchondral bone functions to provide nutrients to articular cartilage, promote the synthesis of protein polysaccharides and collagen fibres, increase the contact surface area, and maintain cartilage. Taken together with the findings of this study, we hypothesize that adiponectin promotes the growth of osteoblasts via the p38 mitogen-activated protein kinase pathway in the subchondral bone of the facet joint that leads to bone remodelling, which results in delayed facet joint degeneration.

### Limitations

There are some limitations in this basic experimental study. In this study, we speculated that adiponectin promotes the growth of osteoblasts via the p38 mitogen-activated protein kinase pathway in the subchondral bone of the facet joint and that this leads to bone remodelling, thereby resulting in delayed facet joint degeneration. However, we have only explored preliminary phenomenon thus far and the specific mechanism involved has not yet been fully determined. Therefore, we will further study the specific mechanism of lumbar facet OA.

## Conclusions

Based on our findings in this study, we hold that adiponectin was expressed in the subchondral bone of the lumbar facet joint and that adiponectin may be inversely correlated with the degree of degeneration of the lumbar facet joint. Therefore, we hypothesize that adiponectin may play a role in protection of the lumbar facet joint degeneration via the modulation of osteoblasts and osteoclasts. Studies are currently underway in our laboratory to further investigate this hypothesis.

## Additional files

Additional file 1: The dataset supporting the conclusions of this article. (XLSX 27 kb)

Additional file 2: Primer design original file. (DOCX 44 kb)

## Abbreviations

BMI: Body mass index
CT: Computed tomography
DG: Degeneration group
ELISA: Enzyme-linked immunosorbent assay
FJOA: Osteoarthritis of the lumbar facet joint
MRI: Magnetic resonance imaging
NG: Normal group
OA: Osteoarthritis
p38 MAPK: p38 mitogen-activated protein kinase
PBS: Phosphate-buffered saline
SDG: Serious degeneration group
TNF: Tumour necrosis factor
