# Supplementary material for: Expression of adiponectin in the subchondral bone of lumbar facet joints with different degrees of degeneration
Source: BMC Musculoskelet Disord. 2017 Nov 3;18:427. doi: 10.1186/s12891-017-1786-8 (PMC5670694; doi:10.1186/s12891-017-1786-8)
Supplement: Supplementary file 2 — Primer design original file. (DOCX 44 kb) [file 12891_2017_1786_MOESM2_ESM.docx]

## Primer pair 1

|  | Sequence (5'->3') | Template strand | Length | Start | Stop | Tm | GC% | Self complementarity | Self 3' complementarity |
| --- | --- | --- | --- | --- | --- | --- | --- | --- | --- |
| Forward primer | AGACATCGGTGAAACCGGAG | Plus | 20 | 369 | 388 | 59.75 | 55.00 | 5.00 | 3.00 |
| Reverse primer | ACACTGAATGCTGAGCGGTA | Minus | 20 | 485 | 466 | 59.39 | 50.00 | 5.00 | 2.00 |
| Product length | 117 | | | | | | | | |

**Products on intended target**

>[XM_011513324.1](http://www.ncbi.nlm.nih.gov/entrez/viewer.fcgi?db=nucleotide&id=767928038) PREDICTED: Homo sapiens adiponectin, C1Q and collagen domain containing (ADIPOQ), transcript variant X1, mRNA

product length = 117

Forward primer 1 AGACATCGGTGAAACCGGAG 20

Template 440 .................... 459

Reverse primer 1 ACACTGAATGCTGAGCGGTA 20

Template 556 .................... 537

>[NM_004797.3](http://www.ncbi.nlm.nih.gov/entrez/viewer.fcgi?db=nucleotide&id=295317373) Homo sapiens adiponectin, C1Q and collagen domain containing (ADIPOQ), transcript variant 2, mRNA

product length = 117

Forward primer 1 AGACATCGGTGAAACCGGAG 20

Template 318 .................... 337

Reverse primer 1 ACACTGAATGCTGAGCGGTA 20

Template 434 .................... 415

>[NM_001177800.1](http://www.ncbi.nlm.nih.gov/entrez/viewer.fcgi?db=nucleotide&id=295317371) Homo sapiens adiponectin, C1Q and collagen domain containing (ADIPOQ), transcript variant 1, mRNA

product length = 117

Forward primer 1 AGACATCGGTGAAACCGGAG 20

Template 369 .................... 388

Reverse primer 1 ACACTGAATGCTGAGCGGTA 20

Template 485 .................... 466

## Primer pair 2

|  | Sequence (5'->3') | Template strand | Length | Start | Stop | Tm | GC% | Self complementarity | Self 3' complementarity |
| --- | --- | --- | --- | --- | --- | --- | --- | --- | --- |
| Forward primer | TGACCAGGAAACCACGACTC | Plus | 20 | 183 | 202 | 59.61 | 55.00 | 3.00 | 3.00 |
| Reverse primer | GGTACTCCGGTTTCACCGAT | Minus | 20 | 392 | 373 | 59.47 | 55.00 | 5.00 | 2.00 |
| Product length | 210 | | | | | | | | |

**Products on intended target**

>[XM_011513324.1](http://www.ncbi.nlm.nih.gov/entrez/viewer.fcgi?db=nucleotide&id=767928038) PREDICTED: Homo sapiens adiponectin, C1Q and collagen domain containing (ADIPOQ), transcript variant X1, mRNA

product length = 210

Forward primer 1 TGACCAGGAAACCACGACTC 20

Template 254 .................... 273

Reverse primer 1 GGTACTCCGGTTTCACCGAT 20

Template 463 .................... 444

>[NM_004797.3](http://www.ncbi.nlm.nih.gov/entrez/viewer.fcgi?db=nucleotide&id=295317373) Homo sapiens adiponectin, C1Q and collagen domain containing (ADIPOQ), transcript variant 2, mRNA

product length = 210

Forward primer 1 TGACCAGGAAACCACGACTC 20

Template 132 .................... 151

Reverse primer 1 GGTACTCCGGTTTCACCGAT 20

Template 341 .................... 322

>[NM_001177800.1](http://www.ncbi.nlm.nih.gov/entrez/viewer.fcgi?db=nucleotide&id=295317371) Homo sapiens adiponectin, C1Q and collagen domain containing (ADIPOQ), transcript variant 1, mRNA

product length = 210

Forward primer 1 TGACCAGGAAACCACGACTC 20

Template 183 .................... 202

Reverse primer 1 GGTACTCCGGTTTCACCGAT 20

Template 392 .................... 373

## Primer pair 3

|  | Sequence (5'->3') | Template strand | Length | Start | Stop | Tm | GC% | Self complementarity | Self 3' complementarity |
| --- | --- | --- | --- | --- | --- | --- | --- | --- | --- |
| Forward primer | CATGCCCATTCGCTTTACCA | Plus | 20 | 516 | 535 | 58.90 | 50.00 | 4.00 | 0.00 |
| Reverse primer | GGAGGCCTGGTCCACATTAT | Minus | 20 | 729 | 710 | 59.15 | 55.00 | 6.00 | 3.00 |
| Product length | 214 | | | | | | | | |

**Products on intended target**

>[XM_011513324.1](http://www.ncbi.nlm.nih.gov/entrez/viewer.fcgi?db=nucleotide&id=767928038) PREDICTED: Homo sapiens adiponectin, C1Q and collagen domain containing (ADIPOQ), transcript variant X1, mRNA

product length = 214

Forward primer 1 CATGCCCATTCGCTTTACCA 20

Template 587 .................... 606

Reverse primer 1 GGAGGCCTGGTCCACATTAT 20

Template 800 .................... 781

>[NM_004797.3](http://www.ncbi.nlm.nih.gov/entrez/viewer.fcgi?db=nucleotide&id=295317373) Homo sapiens adiponectin, C1Q and collagen domain containing (ADIPOQ), transcript variant 2, mRNA

product length = 214

Forward primer 1 CATGCCCATTCGCTTTACCA 20

Template 465 .................... 484

Reverse primer 1 GGAGGCCTGGTCCACATTAT 20

Template 678 .................... 659

>[NM_001177800.1](http://www.ncbi.nlm.nih.gov/entrez/viewer.fcgi?db=nucleotide&id=295317371) Homo sapiens adiponectin, C1Q and collagen domain containing (ADIPOQ), transcript variant 1, mRNA

product length = 214

Forward primer 1 CATGCCCATTCGCTTTACCA 20

Template 516 .................... 535

Reverse primer 1 GGAGGCCTGGTCCACATTAT 20

Template 729 .................... 710

## Primer pair 4

|  | Sequence (5'->3') | Template strand | Length | Start | Stop | Tm | GC% | Self complementarity | Self 3' complementarity |
| --- | --- | --- | --- | --- | --- | --- | --- | --- | --- |
| Forward primer | ATGACCAGGAAACCACGACTC | Plus | 21 | 182 | 202 | 60.00 | 52.38 | 3.00 | 3.00 |
| Reverse primer | CCGATGTCTCCCTTAGGACC | Minus | 20 | 377 | 358 | 58.96 | 60.00 | 5.00 | 3.00 |
| Product length | 196 | | | | | | | | |

**Products on intended target**

>[XM_011513324.1](http://www.ncbi.nlm.nih.gov/entrez/viewer.fcgi?db=nucleotide&id=767928038) PREDICTED: Homo sapiens adiponectin, C1Q and collagen domain containing (ADIPOQ), transcript variant X1, mRNA

product length = 196

Forward primer 1 ATGACCAGGAAACCACGACTC 21

Template 253 ..................... 273

Reverse primer 1 CCGATGTCTCCCTTAGGACC 20

Template 448 .................... 429

>[NM_004797.3](http://www.ncbi.nlm.nih.gov/entrez/viewer.fcgi?db=nucleotide&id=295317373) Homo sapiens adiponectin, C1Q and collagen domain containing (ADIPOQ), transcript variant 2, mRNA

product length = 196

Forward primer 1 ATGACCAGGAAACCACGACTC 21

Template 131 ..................... 151

Reverse primer 1 CCGATGTCTCCCTTAGGACC 20

Template 326 .................... 307

>[NM_001177800.1](http://www.ncbi.nlm.nih.gov/entrez/viewer.fcgi?db=nucleotide&id=295317371) Homo sapiens adiponectin, C1Q and collagen domain containing (ADIPOQ), transcript variant 1, mRNA

product length = 196

Forward primer 1 ATGACCAGGAAACCACGACTC 21

Template 182 ..................... 202

Reverse primer 1 CCGATGTCTCCCTTAGGACC 20

Template 377 .................... 358

# **Homo sapiens adiponectin, C1Q and collagen domain containing (ADIPOQ), transcript variant 1, mRNA**

NCBI Reference Sequence: NM_001177800.1

[FASTA](http://www.ncbi.nlm.nih.gov/nuccore/295317371?report=fasta)聽[Graphics](http://www.ncbi.nlm.nih.gov/nuccore/295317371?report=graph)

[Go to:](http://www.ncbi.nlm.nih.gov/nucleotide/295317371#goto295317371_0)

LOCUS NM_001177800 4629 bp mRNA linear PRI 31-DEC-2015

DEFINITION Homo sapiens adiponectin, C1Q and collagen domain containing

(ADIPOQ), transcript variant 1, mRNA.

ACCESSION NM_001177800

VERSION NM_001177800.1 GI:295317371

KEYWORDS RefSeq.

SOURCE Homo sapiens (human)

ORGANISM [Homo sapiens](http://www.ncbi.nlm.nih.gov/Taxonomy/Browser/wwwtax.cgi?id=9606)

Eukaryota; Metazoa; Chordata; Craniata; Vertebrata; Euteleostomi;

Mammalia; Eutheria; Euarchontoglires; Primates; Haplorrhini;

Catarrhini; Hominidae; Homo.

REFERENCE 1 (bases 1 to 4629)

AUTHORS Ye E, Yang H, Chen L, Chen Q, Sun M, Lin Z, Yu L, Peng M, Zhang C

and Lu X.

TITLE Adiponectin and peroxisome proliferator-activated receptor-gamma

gene polymorphisms and gene-gene interactions with type 2 diabetes

JOURNAL Life Sci. 98 (1), 55-59 (2014)

PUBMED [24440313](http://www.ncbi.nlm.nih.gov/pubmed/24440313)

REMARK GeneRIF: Adiponectin SNP11377 and SNP276 gene-gene interactions are

associated with the increased risk of type 2 diabetes in the Han

people of the Wenzhou region of China

REFERENCE 2 (bases 1 to 4629)

AUTHORS Arikoglu,H., Ozdemir,H., Kaya,D.E., Ipekci,S.H., Arslan,A.,

Kayis,S.A. and Gonen,M.S.

TITLE The Adiponectin variants contribute to the genetic background of

type 2 diabetes in Turkish population

JOURNAL Gene 534 (1), 10-16 (2014)

PUBMED [24404592](http://www.ncbi.nlm.nih.gov/pubmed/24404592)

REMARK GeneRIF: Adiponectin gene polymorphisms might be effective on

susceptibility for type 2 diabetes development which emerged from

the interactions between multiple genes, variants and environmental

factors

REFERENCE 3 (bases 1 to 4629)

AUTHORS Suriyaprom K, Phonrat B and Tungtrongchitr R.

TITLE Association of adiponectin gene -11377C>G polymorphism with

adiponectin levels and the metabolic syndrome in Thais

JOURNAL Asia Pac J Clin Nutr 23 (1), 167-173 (2014)

PUBMED [24561985](http://www.ncbi.nlm.nih.gov/pubmed/24561985)

REMARK GeneRIF: adiponectin gene-11377C>G polymorphism was related to the

metabolic syndrome susceptibility, and this polymorphism impacted

on circulating adiponectin concentrations among Thais

REFERENCE 4 (bases 1 to 4629)

AUTHORS Itoh H, Kawano Y, Furukawa Y, Matsumoto H, Yuge A and Narahara H.

TITLE The role of serum adiponectin levels in women with polycystic

ovarian syndrome

JOURNAL Clin Exp Obstet Gynecol 40 (4), 531-535 (2013)

PUBMED [24597249](http://www.ncbi.nlm.nih.gov/pubmed/24597249)

REMARK GeneRIF: data suggests that adiponectin may play an important role

in the pathogenesis of polycystic ovarian syndrome

REFERENCE 5 (bases 1 to 4629)

AUTHORS Schaffler A, Orso E, Palitzsch KD, Buchler C, Drobnik W, Furst A,

Scholmerich J and Schmitz G.

TITLE The human apM-1, an adipocyte-specific gene linked to the family of

TNF's and to genes expressed in activated T cells, is mapped to

chromosome 1q21.3-q23, a susceptibility locus identified for

familial combined hyperlipidaemia (FCH)

JOURNAL Biochem. Biophys. Res. Commun. 260 (2), 416-425 (1999)

PUBMED [10403784](http://www.ncbi.nlm.nih.gov/pubmed/10403784)

REFERENCE 6 (bases 1 to 4629)

AUTHORS Schaffler A, Langmann T, Palitzsch KD, Scholmerich J and Schmitz G.

TITLE Identification and characterization of the human adipocyte apM-1

promoter

JOURNAL Biochim. Biophys. Acta 1399 (2-3), 187-197 (1998)

PUBMED [9765595](http://www.ncbi.nlm.nih.gov/pubmed/9765595)

REFERENCE 7 (bases 1 to 4629)

AUTHORS Nakano Y, Tobe T, Choi-Miura NH, Mazda T and Tomita M.

TITLE Isolation and characterization of GBP28, a novel gelatin-binding

protein purified from human plasma

JOURNAL J. Biochem. 120 (4), 803-812 (1996)

PUBMED [8947845](http://www.ncbi.nlm.nih.gov/pubmed/8947845)

REFERENCE 8 (bases 1 to 4629)

AUTHORS Hu E, Liang P and Spiegelman BM.

TITLE AdipoQ is a novel adipose-specific gene dysregulated in obesity

JOURNAL J. Biol. Chem. 271 (18), 10697-10703 (1996)

PUBMED [8631877](http://www.ncbi.nlm.nih.gov/pubmed/8631877)

REFERENCE 9 (bases 1 to 4629)

AUTHORS Maeda K, Okubo K, Shimomura I, Funahashi T, Matsuzawa Y and

Matsubara K.

TITLE cDNA cloning and expression of a novel adipose specific

collagen-like factor, apM1 (AdiPose Most abundant Gene transcript

1)

JOURNAL Biochem. Biophys. Res. Commun. 221 (2), 286-289 (1996)

PUBMED [8619847](http://www.ncbi.nlm.nih.gov/pubmed/8619847)

REFERENCE 10 (bases 1 to 4629)

AUTHORS Scherer PE, Williams S, Fogliano M, Baldini G and Lodish HF.

TITLE A novel serum protein similar to C1q, produced exclusively in

adipocytes

JOURNAL J. Biol. Chem. 270 (45), 26746-26749 (1995)

PUBMED [7592907](http://www.ncbi.nlm.nih.gov/pubmed/7592907)COMMENT REVIEWED [REFSEQ](http://www.ncbi.nlm.nih.gov/RefSeq/): This record has been curated by NCBI staff. The

reference sequence was derived from [AC112907.7](http://www.ncbi.nlm.nih.gov/nuccore/22024348), [AK312868.1](http://www.ncbi.nlm.nih.gov/nuccore/164691672) and

[AC072018.6](http://www.ncbi.nlm.nih.gov/nuccore/23343669).

This sequence is a reference standard in the [RefSeqGene](http://www.ncbi.nlm.nih.gov/refseq/rsg/) project.

Summary: This gene is expressed in adipose tissue exclusively. It

encodes a protein with similarity to collagens X and VIII and

complement factor C1q. The encoded protein circulates in the plasma

and is involved with metabolic and hormonal processes. Mutations in

this gene are associated with adiponectin deficiency. Multiple

alternatively spliced variants, encoding the same protein, have

been identified. [provided by RefSeq, Apr 2010].

Transcript Variant: This variant (1) represents the longer

transcript. Both variants 1 and 2 encode the same protein.

Sequence Note: This RefSeq record was created from transcript and

genomic sequence data to make the sequence consistent with the

reference genome assembly. The genomic coordinates used for the

transcript record were based on transcript alignments.

Publication Note: This RefSeq record includes a subset of the

publications that are available for this gene. Please see the Gene

record to access additional publications.

##Evidence-Data-START##

Transcript exon combination :: AK312868.1, DB222528.1 [ECO:0000332]

##Evidence-Data-END##

COMPLETENESS: complete on the 3' end.

PRIMARY REFSEQ_SPAN PRIMARY_IDENTIFIER PRIMARY_SPAN COMP

1-30 AC112907.7 6835-6864 c

31-658 AK312868.1 1-628

659-665 AC072018.6 7019-7025

666-870 AK312868.1 636-840

871-4629 AC072018.6 7231-10989FEATURES Location/Qualifiers source 1..4629

/organism="Homo sapiens"

/mol_type="mRNA"

/db_xref="taxon:[9606](http://www.ncbi.nlm.nih.gov/Taxonomy/Browser/wwwtax.cgi?id=9606)"

/chromosome="3"

/map="3q27" [gene](http://www.ncbi.nlm.nih.gov/nuccore/295317371?from=1&to=4629&sat=4&sat_key=156054305) 1..4629

/gene="ADIPOQ"

/gene_synonym="ACDC; ACRP30; ADIPQTL1; ADPN; APM-1; APM1;

GBP28"

/note="adiponectin, C1Q and collagen domain containing"

/db_xref="GeneID:[9370](http://www.ncbi.nlm.nih.gov/sites/entrez?db=gene&cmd=Retrieve&dopt=full_report&list_uids=9370)"

/db_xref="HGNC:[HGNC:13633](http://www.genenames.org/cgi-bin/gene_symbol_report?hgnc_id=HGNC:13633)"

/db_xref="MIM:[605441](http://www.ncbi.nlm.nih.gov/omim/605441)" [exon](http://www.ncbi.nlm.nih.gov/nuccore/295317371?from=1&to=76&sat=4&sat_key=156054305) 1..76

/gene="ADIPOQ"

/gene_synonym="ACDC; ACRP30; ADIPQTL1; ADPN; APM-1; APM1;

GBP28"

/inference="alignment:Splign:1.39.8" [STS](http://www.ncbi.nlm.nih.gov/nuccore/295317371?from=76&to=1670&sat=4&sat_key=156054305) 76..1670

/gene="ADIPOQ"

/gene_synonym="ACDC; ACRP30; ADIPQTL1; ADPN; APM-1; APM1;

GBP28"

/standard_name="L17971"

/db_xref="UniSTS:[43966](http://www.ncbi.nlm.nih.gov/probe?term=43966 [UniSTS ID])" [exon](http://www.ncbi.nlm.nih.gov/nuccore/295317371?from=77&to=127&sat=4&sat_key=156054305) 77..127

/gene="ADIPOQ"

/gene_synonym="ACDC; ACRP30; ADIPQTL1; ADPN; APM-1; APM1;

GBP28"

/inference="alignment:Splign:1.39.8" [exon](http://www.ncbi.nlm.nih.gov/nuccore/295317371?from=128&to=349&sat=4&sat_key=156054305) 128..349

/gene="ADIPOQ"

/gene_synonym="ACDC; ACRP30; ADIPQTL1; ADPN; APM-1; APM1;

GBP28"

/inference="alignment:Splign:1.39.8" [CDS](http://www.ncbi.nlm.nih.gov/nuccore/295317371?from=136&to=870&sat=4&sat_key=156054305) 136..870

/gene="ADIPOQ"

/gene_synonym="ACDC; ACRP30; ADIPQTL1; ADPN; APM-1; APM1;

GBP28"

/note="gelatin-binding protein 28; adipose specific

collagen-like factor; 30 kDa adipocyte complement-related

protein; adipocyte complement-related 30 kDa protein;

adipose most abundant gene transcript 1 protein"

/codon_start=1

/product="adiponectin precursor"

/protein_id="[NP_001171271.1](http://www.ncbi.nlm.nih.gov/protein/295317372)"

/db_xref="GI:295317372"

/db_xref="CCDS:[CCDS3284.1](http://www.ncbi.nlm.nih.gov/CCDS/CcdsBrowse.cgi?REQUEST=CCDS&DATA=CCDS3284.1)"

/db_xref="GeneID:[9370](http://www.ncbi.nlm.nih.gov/sites/entrez?db=gene&cmd=Retrieve&dopt=full_report&list_uids=9370)"

/db_xref="HGNC:[HGNC:13633](http://www.genenames.org/cgi-bin/gene_symbol_report?hgnc_id=HGNC:13633)"

/db_xref="MIM:[605441](http://www.ncbi.nlm.nih.gov/omim/605441)"

/translation="MLLLGAVLLLLALPGHDQETTTQGPGVLLPLPKGACTGWMAGIP

GHPGHNGAPGRDGRDGTPGEKGEKGDPGLIGPKGDIGETGVPGAEGPRGFPGIQGRKG

EPGEGAYVYRSAFSVGLETYVTIPNMPIRFTKIFYNQQNHYDGSTGKFHCNIPGLYYF

AYHITVYMKDVKVSLFKKDKAMLFTYDQYQENNVDQASGSVLLHLEVGDQVWLQVYGE

GERNGLYADNDNDSTFTGFLLYHDTN" [sig_peptide](http://www.ncbi.nlm.nih.gov/nuccore/295317371?from=136&to=177&sat=4&sat_key=156054305) 136..177

/gene="ADIPOQ"

/gene_synonym="ACDC; ACRP30; ADIPQTL1; ADPN; APM-1; APM1;

GBP28" [mat_peptide](http://www.ncbi.nlm.nih.gov/nuccore/295317371?from=178&to=867&sat=4&sat_key=156054305) 178..867

/gene="ADIPOQ"

/gene_synonym="ACDC; ACRP30; ADIPQTL1; ADPN; APM-1; APM1;

GBP28"

/product="adiponectin" [misc_feature](http://www.ncbi.nlm.nih.gov/nuccore/295317371?from=265&to=267&sat=4&sat_key=156054305) 265..267

/gene="ADIPOQ"

/gene_synonym="ACDC; ACRP30; ADIPQTL1; ADPN; APM-1; APM1;

GBP28"

/experiment="experimental evidence, no additional details

recorded"

/note="4-hydroxyproline; propagated from

UniProtKB/Swiss-Prot (Q15848.1); hydroxylation site" [misc_feature](http://www.ncbi.nlm.nih.gov/nuccore/295317371?from=274&to=276&sat=4&sat_key=156054305) 274..276

/gene="ADIPOQ"

/gene_synonym="ACDC; ACRP30; ADIPQTL1; ADPN; APM-1; APM1;

GBP28"

/experiment="experimental evidence, no additional details

recorded"

/note="4-hydroxyproline; propagated from

UniProtKB/Swiss-Prot (Q15848.1); hydroxylation site" [misc_feature](http://www.ncbi.nlm.nih.gov/nuccore/295317371?from=292&to=294&sat=4&sat_key=156054305) 292..294

/gene="ADIPOQ"

/gene_synonym="ACDC; ACRP30; ADIPQTL1; ADPN; APM-1; APM1;

GBP28"

/experiment="experimental evidence, no additional details

recorded"

/note="4-hydroxyproline; propagated from

UniProtKB/Swiss-Prot (Q15848.1); hydroxylation site" [misc_feature](http://www.ncbi.nlm.nih.gov/nuccore/295317371?from=319&to=321&sat=4&sat_key=156054305) 319..321

/gene="ADIPOQ"

/gene_synonym="ACDC; ACRP30; ADIPQTL1; ADPN; APM-1; APM1;

GBP28"

/experiment="experimental evidence, no additional details

recorded"

/note="Not hydroxylated; propagated from

UniProtKB/Swiss-Prot (Q15848.1); other site" [misc_feature](http://www.ncbi.nlm.nih.gov/nuccore/295317371?from=328&to=330&sat=4&sat_key=156054305) 328..330

/gene="ADIPOQ"

/gene_synonym="ACDC; ACRP30; ADIPQTL1; ADPN; APM-1; APM1;

GBP28"

/experiment="experimental evidence, no additional details

recorded"

/note="5-hydroxylysine; propagated from

UniProtKB/Swiss-Prot (Q15848.1); hydroxylation site" [misc_feature](http://www.ncbi.nlm.nih.gov/nuccore/295317371?from=337&to=339&sat=4&sat_key=156054305) 337..339

/gene="ADIPOQ"

/gene_synonym="ACDC; ACRP30; ADIPQTL1; ADPN; APM-1; APM1;

GBP28"

/experiment="experimental evidence, no additional details

recorded"

/note="5-hydroxylysine; propagated from

UniProtKB/Swiss-Prot (Q15848.1); hydroxylation site" [misc_feature](http://www.ncbi.nlm.nih.gov/nuccore/295317371?from=346&to=348&sat=4&sat_key=156054305) 346..348

/gene="ADIPOQ"

/gene_synonym="ACDC; ACRP30; ADIPQTL1; ADPN; APM-1; APM1;

GBP28"

/experiment="experimental evidence, no additional details

recorded"

/note="4-hydroxyproline, partial; propagated from

UniProtKB/Swiss-Prot (Q15848.1); hydroxylation site" [misc_feature](http://www.ncbi.nlm.nih.gov/nuccore/295317371?from=361&to=363&sat=4&sat_key=156054305) 361..363

/gene="ADIPOQ"

/gene_synonym="ACDC; ACRP30; ADIPQTL1; ADPN; APM-1; APM1;

GBP28"

/experiment="experimental evidence, no additional details

recorded"

/note="4-hydroxyproline, partial; propagated from

UniProtKB/Swiss-Prot (Q15848.1); hydroxylation site" [misc_feature](http://www.ncbi.nlm.nih.gov/nuccore/295317371?from=364&to=366&sat=4&sat_key=156054305) 364..366

/gene="ADIPOQ"

/gene_synonym="ACDC; ACRP30; ADIPQTL1; ADPN; APM-1; APM1;

GBP28"

/experiment="experimental evidence, no additional details

recorded"

/note="5-hydroxylysine; propagated from

UniProtKB/Swiss-Prot (Q15848.1); hydroxylation site" [misc_feature](http://www.ncbi.nlm.nih.gov/nuccore/295317371?from=391&to=393&sat=4&sat_key=156054305) 391..393

/gene="ADIPOQ"

/gene_synonym="ACDC; ACRP30; ADIPQTL1; ADPN; APM-1; APM1;

GBP28"

/experiment="experimental evidence, no additional details

recorded"

/note="Not hydroxylated; propagated from

UniProtKB/Swiss-Prot (Q15848.1); other site" [misc_feature](http://www.ncbi.nlm.nih.gov/nuccore/295317371?from=406&to=408&sat=4&sat_key=156054305) 406..408

/gene="ADIPOQ"

/gene_synonym="ACDC; ACRP30; ADIPQTL1; ADPN; APM-1; APM1;

GBP28"

/experiment="experimental evidence, no additional details

recorded"

/note="4-hydroxyproline; propagated from

UniProtKB/Swiss-Prot (Q15848.1); hydroxylation site" [misc_feature](http://www.ncbi.nlm.nih.gov/nuccore/295317371?from=418&to=420&sat=4&sat_key=156054305) 418..420

/gene="ADIPOQ"

/gene_synonym="ACDC; ACRP30; ADIPQTL1; ADPN; APM-1; APM1;

GBP28"

/experiment="experimental evidence, no additional details

recorded"

/note="4-hydroxyproline, partial; propagated from

UniProtKB/Swiss-Prot (Q15848.1); hydroxylation site" [misc_feature](http://www.ncbi.nlm.nih.gov/nuccore/295317371?from=436&to=438&sat=4&sat_key=156054305) 436..438

/gene="ADIPOQ"

/gene_synonym="ACDC; ACRP30; ADIPQTL1; ADPN; APM-1; APM1;

GBP28"

/experiment="experimental evidence, no additional details

recorded"

/note="5-hydroxylysine; propagated from

UniProtKB/Swiss-Prot (Q15848.1); hydroxylation site" [misc_feature](http://www.ncbi.nlm.nih.gov/nuccore/295317371?from=445&to=447&sat=4&sat_key=156054305) 445..447

/gene="ADIPOQ"

/gene_synonym="ACDC; ACRP30; ADIPQTL1; ADPN; APM-1; APM1;

GBP28"

/experiment="experimental evidence, no additional details

recorded"

/note="Not hydroxylated; propagated from

UniProtKB/Swiss-Prot (Q15848.1); other site" [misc_feature](http://www.ncbi.nlm.nih.gov/nuccore/295317371?from=823&to=825&sat=4&sat_key=156054305) 823..825

/gene="ADIPOQ"

/gene_synonym="ACDC; ACRP30; ADIPQTL1; ADPN; APM-1; APM1;

GBP28"

/experiment="experimental evidence, no additional details

recorded"

/note="Not glycosylated; propagated from

UniProtKB/Swiss-Prot (Q15848.1); other site" [exon](http://www.ncbi.nlm.nih.gov/nuccore/295317371?from=350&to=4629&sat=4&sat_key=156054305) 350..4629

/gene="ADIPOQ"

/gene_synonym="ACDC; ACRP30; ADIPQTL1; ADPN; APM-1; APM1;

GBP28"

/inference="alignment:Splign:1.39.8" [STS](http://www.ncbi.nlm.nih.gov/nuccore/295317371?from=880&to=1087&sat=4&sat_key=156054305) 880..1087

/gene="ADIPOQ"

/gene_synonym="ACDC; ACRP30; ADIPQTL1; ADPN; APM-1; APM1;

GBP28"

/standard_name="G29832"

/db_xref="UniSTS:[10094](http://www.ncbi.nlm.nih.gov/probe?term=10094 [UniSTS ID])" [STS](http://www.ncbi.nlm.nih.gov/nuccore/295317371?from=1876&to=2741&sat=4&sat_key=156054305) 1876..2741

/gene="ADIPOQ"

/gene_synonym="ACDC; ACRP30; ADIPQTL1; ADPN; APM-1; APM1;

GBP28"

/standard_name="GDB:434012"

/db_xref="UniSTS:[157204](http://www.ncbi.nlm.nih.gov/probe?term=157204 [UniSTS ID])" [STS](http://www.ncbi.nlm.nih.gov/nuccore/295317371?from=1887&to=2725&sat=4&sat_key=156054305) 1887..2725

/gene="ADIPOQ"

/gene_synonym="ACDC; ACRP30; ADIPQTL1; ADPN; APM-1; APM1;

GBP28"

/standard_name="L17709"

/db_xref="UniSTS:[42599](http://www.ncbi.nlm.nih.gov/probe?term=42599 [UniSTS ID])" [STS](http://www.ncbi.nlm.nih.gov/nuccore/295317371?from=2721&to=4329&sat=4&sat_key=156054305) 2721..4329

/gene="ADIPOQ"

/gene_synonym="ACDC; ACRP30; ADIPQTL1; ADPN; APM-1; APM1;

GBP28"

/standard_name="D8S2279"

/db_xref="UniSTS:[473907](http://www.ncbi.nlm.nih.gov/probe?term=473907 [UniSTS ID])" [STS](http://www.ncbi.nlm.nih.gov/nuccore/295317371?from=4241&to=4329&sat=4&sat_key=156054305) 4241..4329

/gene="ADIPOQ"

/gene_synonym="ACDC; ACRP30; ADIPQTL1; ADPN; APM-1; APM1;

GBP28"

/standard_name="D8S2279"

/db_xref="UniSTS:[473907](http://www.ncbi.nlm.nih.gov/probe?term=473907 [UniSTS ID])" [regulatory](http://www.ncbi.nlm.nih.gov/nuccore/295317371?from=4610&to=4615&sat=4&sat_key=156054305) 4610..4615

/regulatory_class="polyA_signal_sequence"

/gene="ADIPOQ"

/gene_synonym="ACDC; ACRP30; ADIPQTL1; ADPN; APM-1; APM1;

GBP28" [polyA_site](http://www.ncbi.nlm.nih.gov/nuccore/295317371?from=4629&to=4629&sat=4&sat_key=156054305) 4629

/gene="ADIPOQ"

/gene_synonym="ACDC; ACRP30; ADIPQTL1; ADPN; APM-1; APM1;

GBP28"ORIGIN  1 aggctgttga ggctgggcca tctcctcctc acttccattc tgactgcagt ctgtggttct

61 gattccatac cagaggagac gggatttcac catgttgtcc aggctggtct gaaactcctg

121 acatcagggc tcaggatgct gttgctggga gctgttctac tgctattagc tctgcccggt

181 catgaccagg aaaccacgac tcaagggccc ggagtcctgc ttcccctgcc caagggggcc

241 tgcacaggtt ggatggcggg catcccaggg catccgggcc ataatggggc cccaggccgt

301 gatggcagag atggcacccc tggtgagaag ggtgagaaag gagatccagg tcttattggt

361 cctaagggag acatcggtga aaccggagta cccggggctg aaggtccccg aggctttccg

421 ggaatccaag gcaggaaagg agaacctgga gaaggtgcct atgtataccg ctcagcattc

481 agtgtgggat tggagactta cgttactatc cccaacatgc ccattcgctt taccaagatc

541 ttctacaatc agcaaaacca ctatgatggc tccactggta aattccactg caacattcct

601 gggctgtact actttgccta ccacatcaca gtctatatga aggatgtgaa ggtcagcctc

661 ttcaagaagg acaaggctat gctcttcacc tatgatcagt accaggaaaa taatgtggac

721 caggcctccg gctctgtgct cctgcatctg gaggtgggcg accaagtctg gctccaggtg

781 tatggggaag gagagcgtaa tggactctat gctgataatg acaatgactc caccttcaca

841 ggctttcttc tctaccatga caccaactga tcaccactaa ctcagagcct cctccaggcc

901 aaacagcccc aaagtcaatt aaaggctttc agtacggtta ggaagttgat tattatttag

961 ttggaggcct ttagatatta ttcattcatt tactcattca tttattcatt cattcatcga

1021 gtaactttaa aaaaatcata tgctatgttc ccagtcctgg ggagcttcac aaacatgacc

1081 agataactga ctagaaagaa gtagttgaca gtgctatttt gtgcccactg tctctcctga

1141 tgctcatatc aatcctataa ggcacaggga acaagcattc tcctgttttt acagattgta

1201 tcctgaggct gagagagtta agtgaatgtc taaggtcaca cagtattaag tgacagtgct

1261 agaaatcaaa cccagagctg tggactttgt tcactagact gtgccctttt atagaggtac

1321 atgttctctt tggagtgttg gtaggtgtct gtttcccacc tcacctgaga gccattgaat

1381 ttgccttcct catgaattaa aacctccccc aagcagagct tcctcagaga aagtggttct

1441 atgatgacgt cctgtcttgg aaggactact actcaatggc ccctgcacta ctctacttcc

1501 tcttacctat gtcccttctc atgcctttcc ctccaacggg gaaagccaac tccatctcta

1561 agtgccgaac tcatccctgt tcctcaaggc cacctggcca ggagcttctc tgatgtgata

1621 tccacttttt ttttttttga gatggagtct cactctgtca cccaggctgg agtacagtga

1681 cacgacctcg gctcactgca gcctccttct cctgggtcca agcaattatt gtgcctcagc

1741 ctcccgagta gctgagactt caggtgcatt ccaccacaca tggctaattt ttgtattttt

1801 agtagaaatg gggtttcgtc atgttggcca ggctggtctc gaactcctgg cctaggtgat

1861 ccacccgcct cgacctccca aagtgctggg attacaggca tgagccacca tgcccagtcg

1921 atatctcact ttttattttg ccatggatga gagtcctggg tgtgaggaac acctcccacc

1981 aggctagagg caactgccca ggaaggactg tgcttccgtc acctctaaat cccttgcaga

2041 tccttgataa atgcctcatg aagaccaatc tcttgaatcc catatctacc cagaattaac

2101 tccattccag tctctgcatg taatcagttt tatccacaga aacattttca ttttaggaaa

2161 tccctggttt taagtatcaa tccttgttca gctggacaat atgaatcttt tccactgaag

2221 ttagggatga ctgtgatttt cagaacacgt ccagaatttt tcatcaagaa ggtagcttga

2281 gcctgaaatg caaaacccat ggaggaattc tgaagccatt gtctccttga gtaccaacag

2341 ggtcagggaa gactgggcct cctgaattta ttattgttct ttaagaatta caggttgagg

2401 tagttgatgg tggtaaacat tctctcagga gacaataact ccagtgatgt tcttcaaaga

2461 ttttagcaaa aacagagtaa atagcattct ctatcaatat ataaatttaa aaaactatct

2521 ttttgcttac agttttaaat tctgaacaat tctctcttat atgtgtattg ctaatcatta

2581 aggtattatt ttttccacat ataaagcttt gtctttttgt tgttgttgtt gtttttaaga

2641 tggagtttcc ctctgttgcc aggctagagt gcagtggcat gatctcggct tactgcaacc

2701 tttgcctccc aggttcaagc gattcttctg cctcagcctc ccgagtagct gggaccacag

2761 gtgcctacca ccatgccagg ctaatttttg tatttttagt aaagacaggg tttcaccata

2821 ttggccaggc tggtctcgaa ctcctgacct tgtgatctgc ccgcctccat ttttgttgtt

2881 attttttgag aaagatagat atgaggttta gagagggatg aagaggtgag agtaagcctt

2941 gtgttagtca gaactctgtg ttgtgaatgt cattcacaac agaaaaccca aaatattatg

3001 caaactactg taagcaagaa aaataaagga aaaatggaaa catttattcc tttgcataat

3061 agaaattacc agagttgttc tgtctttaga taaggtttga accaaagctc aaaacaatca

3121 agaccctttt ctgtatgtcc ttctgttctg ccttccgcag tgtaggcttt accctcaggt

3181 gctacacagt atagttctag ggtttccctc ccgatatcaa aaagactgtg gcctgcccag

3241 ctctcgtatc cccaagccac accatctggc taaatggaca tcatgttttc tggtgatgcc

3301 caaagaggag agaggaagct ctctttccca gatgccccag caagtgtaac cttgcatctc

3361 attgctctgg ctgagttgtg tgcctgtttc tgaccaatca ctgagtcagg aggatgaaat

3421 attcatattg acttaattgc agcttaagtt aggggtatgt agaggtattt tccctaaagc

3481 aaaattggga cactgttatc agaaatagga gagtggatga tagatgcaaa ataatacctg

3541 tccacaacaa actcttaatg ctgtgtttga gctttcatga gtttcccaga gagacatagc

3601 tggaaaattc ctattgattt tctctaaaat ttcaacaagt agctaaagtc tggctatgct

3661 cacagtctca catctggttg gggtgggctc cttacagaac acgctttcac agttacccta

3721 aactctctgg ggcagggtta ttcctttgtg gaaccagagg cacagagaga gtcaactgag

3781 gccaaaagag gcctgagaga aactgaggtc aagatttcag gattaatggt cctgtgatgc

3841 tttgaagtac aattgtggat ttgtccaatt ctctttagtt ctgtcagctt ttgcttcata

3901 tattttagcg ctctattatt agatatatac atgtttagta ttatgtctta ttggtgcatt

3961 tactctctta tcattatgta atgtccttct ttatctgtga taattttctg tgttctgaag

4021 tctactttgt ctaaaaataa catacgcact caacttcctt ttctttcttc cttcctttct

4081 ttcttccttc ctttctttct ctctctctct ctttccttcc ttccttcctc cttttctttc

4141 tctctctctc tctctctctt tttttgacag actctcgttc tgtggccctg gctggagttc

4201 agtggtgtga tcttggctca ctgctacctc taccatgagc aattctcctg cctcagcctc

4261 ccaagtagct ggaactacag gctcatgcca ctgcgcccag ctaatttttg tatttttcgt

4321 agagacgggg tttcaccaca ttcgtcaggt tggtttcaaa ctcctgactt tgtgatccac

4381 ccgcctcggc ctcccaaagt gctgggatta caggcatgag ccatcacacc tggtcaactt

4441 tcttttgatt agtgtttttg tggtatatct ttttccatca tgttacttta aatatatcta

4501 tattattgta tttaaaatgt gtttcttaca gactgcatgt agttgggtat aatttttatc

4561 cagtctaaaa atatctgtct tttaattggt gtttagacaa tttatattta ataaaattgt

4621 tgaatttaa

//
